# Characterization of the Optical Properties of Turbid Media by Supervised Learning of Scattering Patterns

**DOI:** 10.1038/s41598-017-15601-4

**Published:** 2017-11-10

**Authors:** Iman Hassaninia, Ramin Bostanabad, Wei Chen, Hooman Mohseni

**Affiliations:** 1 Department of Electrical Engineering and Computer Science, Northwestern University, Evanston, IL 60208 USA; 2 Department of Mechanical Engineering, Northwestern University, Evanston, IL 60208 USA

## Abstract

Fabricated tissue phantoms are instrumental in optical *in-vitro* investigations concerning cancer diagnosis, therapeutic applications, and drug efficacy tests. We present a simple non-invasive computational technique that, when coupled with experiments, has the potential for characterization of a wide range of biological tissues. The fundamental idea of our approach is to find a supervised learner that links the scattering pattern of a turbid sample to its thickness and scattering parameters. Once found, this supervised learner is employed in an inverse optimization problem for estimating the scattering parameters of a sample given its thickness and scattering pattern. Multi-response Gaussian processes are used for the supervised learning task and a simple setup is introduced to obtain the scattering pattern of a tissue sample. To increase the predictive power of the supervised learner, the scattering patterns are filtered, enriched by a regressor, and finally characterized with two parameters, namely, transmitted power and scaled Gaussian width. We computationally illustrate that our approach achieves errors of roughly 5% in predicting the scattering properties of many biological tissues. Our method has the potential to facilitate the characterization of tissues and fabrication of phantoms used for diagnostic and therapeutic purposes over a wide range of optical spectrum.

## Introduction

Recently, considerable effort has been devoted to improving the quality of fabricated tissue phantoms^1–4^ as they are instrumental in the optical *in-vitro* investigations concerning cancer diagnosis^5^, therapeutic applications^6,7^, and drug efficacy tests^8^. In this regard, one avenue of research has pursued the use of accurate and cost-effective phantom characterization techniques to guide the fabrication process. The most widely recognized characterization techniques for this purpose are spatial frequency domain imaging (SFDI)^9,10^, frequency domain photon migration (FDPM)^11,12^, and inverse adding-doubling (IAD)^13,14^. The fundamental idea of these techniques is to computationally model the scattering phenomenon in tissue phantoms and subsequently estimate the scattering properties of such materials by calibrating the computational model against some experimental data. Below, we briefly describe these methods and then introduce our approach which enables the data-driven estimation of scattering properties of tissues by employing a supervised learner (which is fitted to a training dataset of tissues’ characteristics) in an inverse optimization procedure. Our method is inexpensive, non-intrusive, efficient, and applicable to a wide range of materials.

In the case of a wavefront with a lateral sinusoidal intensity profile, the penetration depth and the diffuse reflectance depend on the lateral spatial frequency. The latter quantity, can be used to obtain the optical properties as well as the optical tomography of the sample^10^. The essence of the SFDI technique is to employ this relation by matching the measured and calculated diffuse reflectance for a set of wavefronts with different spatial frequencies. As for the FDPM technique, the analytical expressions of the phase lag, amplitude attenuation, and complex wave vector of a semi-infinite turbid medium are fitted to the corresponding measured values of the reflected beam to find the scattering parameters. In FDPM, the two ways of collecting the fitting data are: $(i)$ for a fixed temporal modulation frequency, the distance between the LED source and the detector is changed, and $(ii)$ for a fixed distance between the source and the detector, the temporal modulation frequency is varied over a wide range.

In both SFDI and FDPM methods, the diffusion equation is used to approximate the Boltzmann transport equation. This results in the overestimation (underestimation) of the diffuse reflectance at low (high) spatial frequencies. In addition to the fitting error, enforcing the boundary conditions in the diffusion equation^15^ introduces some error in arriving at the analytical formulas for realistic semi-infinite media. Moreover, the experimental setup in both SFDI and FDPM methods are complex and costly. In SFDI, in addition to a spatial light modulator, two polarizers at the source and detector are needed to reject the specular reflection collected normal to the surface. As for FDPM technique, a network analyzer is required to modulate the current of the LED and to detect the diffused reflectance of the temporally modulated beam. These instruments render the setup complex and costly. Furthermore, these methods are incapable of measuring the *anisotropy coefficient* of the sample, $g$, which is an important parameter for characterizing turbid media^16–18^. In biological tissues, the probability of scattering a beam of light at an angle $\theta$ (with respect to the incoming beam) can be described suitably by the Henyey-Greenstein phase function^19,20^:

1 $$P(\theta )=\frac{1}{4\pi }\frac{(1-{g}^{2})}{{(1+{g}^{2}-2{gcos}(\theta ))}^{\frac{3}{2}}},$$

where the optical properties of the turbid medium depend on both $g$ (that characterizes the angular profile of scattering) as well as the scattering length, ${s}_{l}$, the average distance over which the scattering occurs.

Among these techniques, IAD is the most popular one due to its relatively higher accuracy and simpler experimental setup. Briefly, IAD is based on matching the measured and the calculated diffuse reflectance and transmittance by calibrating the scattering and absorption coefficients used in the simulations. When an accurate measurement of the un-scattered transmission can be made, it is possible to obtain $g$ as well. In IAD, the errors are mostly attributed to the experimental data. For instance, when measuring the total transmission and reflectance, part of the light scattered from the edge of the sample can be lost, or when measuring the un-scattered transmission, the scattered rays may unavoidably influence the measurement^13^.

We propose an efficient method to address the above challenges and have a better compromise between accuracy and the cost of measuring the scattering parameters (i.e., $g$ and ${s}_{l}$). Our method is based on a supervised learner that can predict the scattering pattern of a turbid medium given its thickness ($t$) and scattering parameters. Once this supervised learner is found, the scattering parameters of any turbid sample can be calculated given its thickness and the image of the scattered rays’ pattern either by inversing the supervised learner or performing an optimization task.

**Figure 1:**
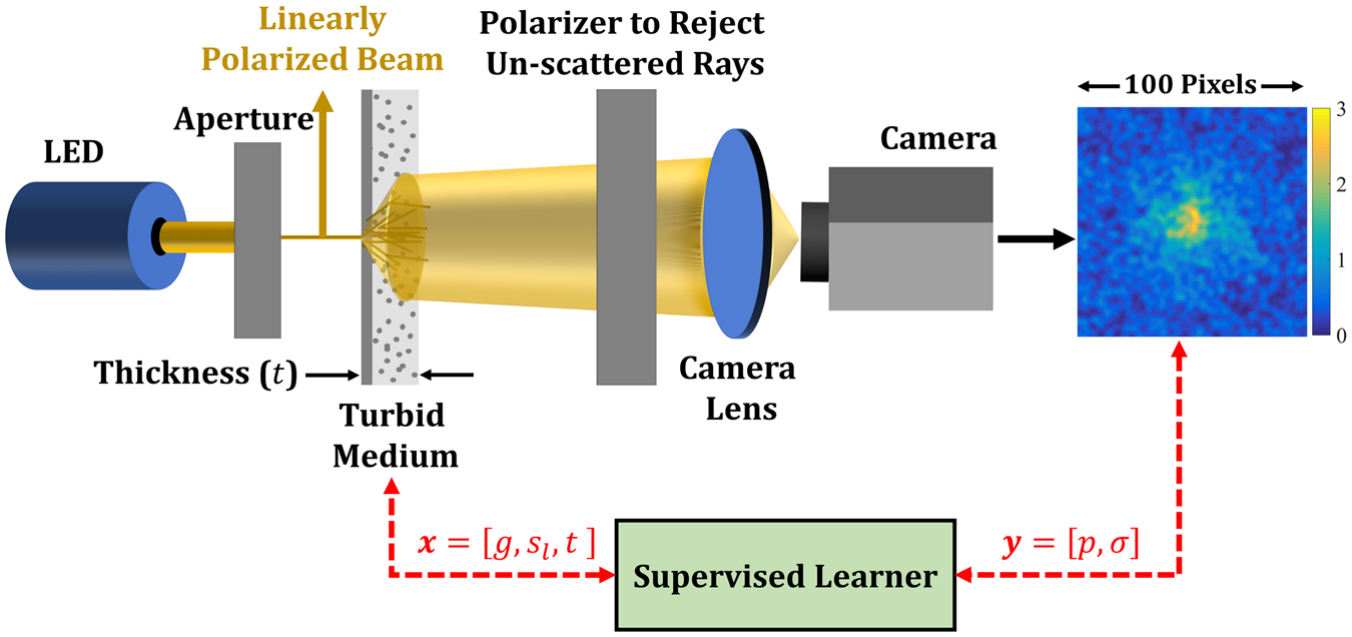
The schematics of the simulation setup: The image of the transmitted scattered waves on the surface of the turbid medium is used to obtain its scattering properties. The input to the turbid medium is a linearly polarized pencil beam of an LED. A free space polarizer is used to reject the un-scattered beam based on the polarization of the input LED. The color bar besides the image indicates the range of gray intensity. See the text for more details on our simulation setup.

Our process for obtaining the scattering pattern, as illustrated in Fig. 1, starts by producing a pencil beam from an LED placed behind an aperture. The pencil beam has a well-defined but arbitrary polarization and is incident on the turbid medium with a *known* thickness. The surface of this medium is then imaged to a camera sensor through a lens, where the un-scattered beam with the well-defined polarization is rejected via a polarizer placed next to the turbid medium. We note that with such a non-coherent and phase-insensitive measurement, the size of the image as well as the components scale with the dimeter of the laser. Because of this scaling rule, the length unit of the image shown in Fig. 1 equals the number of the *scaled* pixels of the camera. We also note that for a collimated illumination, the distance between the source and the sample is arbitrary. A similar argument holds for the distance between the polarizer and the sample because the un-scattered light is collimated.

We employed the same configuration as in Fig. 1 in our computational simulations. In particular, we placed the camera lens far from the sample ($15\,cm)$ such that the scattered light is almost parallel to the optical axis. We employed a lens with a focal length, radius, and maximum numerical aperture of, respectively, $4\,cm$, $6\,mm$, and $0.15$. Additionally, the optical resolution of the system according to Rayleigh’s criterion was $4\,\mu m$ at $1550\,nm$ ^21^, which is equal to the pixel pitch of the detector in our simulations. Since the pixel pitch was larger than half of the optical resolving limit and hence the Nyquist criterion was not satisfied, the scattering patterns are slightly blurred. As for the LED bandwidth, Δ*λ*, we chose it wide enough to have a coherent length much smaller than the optical path length of the rays (${L}_{coh}\, < 0.1{L}_{opt}$). With this choice, the coherent effects do not distort the scattered images. In particular, we ensured that Δ*λ* $\, > \,\frac{2\,\mathrm{ln}(2)}{\pi n}\frac{{\lambda }^{2}}{0.1{L}_{opt}}$ where *n* is the refractive index and $\lambda$ is the wavelength of interest. The minimum required bandwidth is $40\,nm$ when ${L}_{opt}=200\,\mu m$, $n=1.33$, and $\lambda =1550\,nm$.

We note that, in our method the simulations are performed on thin slabs of phantom or tissue with known thickness. Although performing the same type of experiment using *reflection* is in principle possible, we expect much weaker reflection than transmission for such thin slabs. Additionally, the reduction of the signal strength translates into lower SNR and higher measurement errors in the case of reflection. We have also found some experimental works which are based on quantitative *phase of the transmission* images of thin samples for which $t <  < \,{s}_{l}$ ^22,23^. As opposed to this latter approach, our method is based on the intensity of the scattering patterns which is simpler and applicable to thicker samples.

To fit our supervised learner, a high-fidelity training dataset of input-output pairs is required. Here, the inputs (collectively denoted by ${\boldsymbol{x}}$) are the characteristics of the turbid samples (i.e., $g$, ${s}_{l}$, and $t$) while the outputs (collectively denoted by ${\boldsymbol{y}}$) are some finite set of parameters that characterize the corresponding scattering patterns (i.e., the images similar to the one in Fig. 1). We elaborate on the choice of the latter parameters in Sec. 0 but note that they must be sufficiently robust to noise so that, given $t$, the scattering parameters of any turbid sample can be predicted with relatively high accuracy using the supervised learner.

## Results

To construct the computational training dataset, we used the Sobol sequence^24,25^ to build a space filling design of experiments (DOE) with $400$. points (i.e., simulation settings) over the hypercube $0.7\le g\le 0.93\,$, $0.03\le {s}_{l}\le 0.12\,mm$, and $200\le t\le 600\,\mu m$. It is noted that the lower limit on the sample thickness is because of the considerable inaccuracies associated with the negligible probability of scattering in thin samples. In contrast, the upper limit on the sample thickness is bounded due to the computational costs associated with tracing the large number of ray scatterings. As for ranges of *g* and ${s}_{l}$, they cover the scattering properties of a wide range of biological tissues including but not limited to liver^26^, white brain matter, grey brain matter, cerebellum, and brainstem tissues (pons, thalamus)^27^.

Once the simulation settings were determined, following the schematic in Fig. 1, the scattering pattern corresponding to each of them was obtained by the commercial raytracing software *Zemax OpticStudio*. Although there are many software programs applicable for this task (such as *Code v*, *Oslo*, and *FRED*), *Zemax* is perhaps the most widely used software for ray tracing. Unlike mode solvers, ray tracing is computationally fast. The significant scattering effects as well as the employed broadband light source (i.e., the LED) further justify the use of a ray-tracing software. In our simulations with *Zemax* , rigorous Monte-Carlo simulations were conducted for higher accuracy (instead of solving the simplified diffusion equation) and the turbid media were simulated with the built-in Henyey-Greenstein model^28^. To push the upper limit on the sample thickness to 600 *μm*, we increased the number of Monte Carlo intersections and observed that the maximum capacity of *Zemax* (roughly two million segments per ray) must be employed for sufficient accuracy. Additionally, we found that a 100 × 100 rectangular detector and five million launched rays provide a reasonable compromise between the accuracy and the simulation costs (about 3 minutes for each input setting).

As mentioned in Sec. 1, the scattering patterns corresponding to the simulation settings (i.e., the DOE points) must be characterized with a finite set of parameters (denoted by ***y*** in Fig. 1) to reduce the problem dimensionality and enable the supervised learning process. To determine the sufficient number of parameters, we highlight that our end goal is to arrive at an inverse relation where the *g* and ${s}_{l}$ of a tissue sample with a *specific* thickness can be predicted. Therefore, if the parameters are chosen such that both *g* and ${s}_{l}$ are monotonic functions of them, two characterizing parameters are required for a one-to-one relation. It must be noted that, these parameters must be sufficiently robust to the inherent errors in the simulations mentioned above. We will elaborate on this latter point below and in Sec. 3.

We have conducted extensive studies and our results indicate that the *transmitted power*, *p*, and the *scaled Gaussian width*, *σ*, can sufficiently and robustly characterize the scattering patterns of a wide range of tissue samples. While *p* measures the amount of the LED beam power transmitted through the sample and collected at the image, *σ* measures the extent to which the sample scatters the LED beam. It is evident that these parameters are negatively correlated, i.e., increasing *p* would decrease *σ* and vice versa.

**Figure 2:**
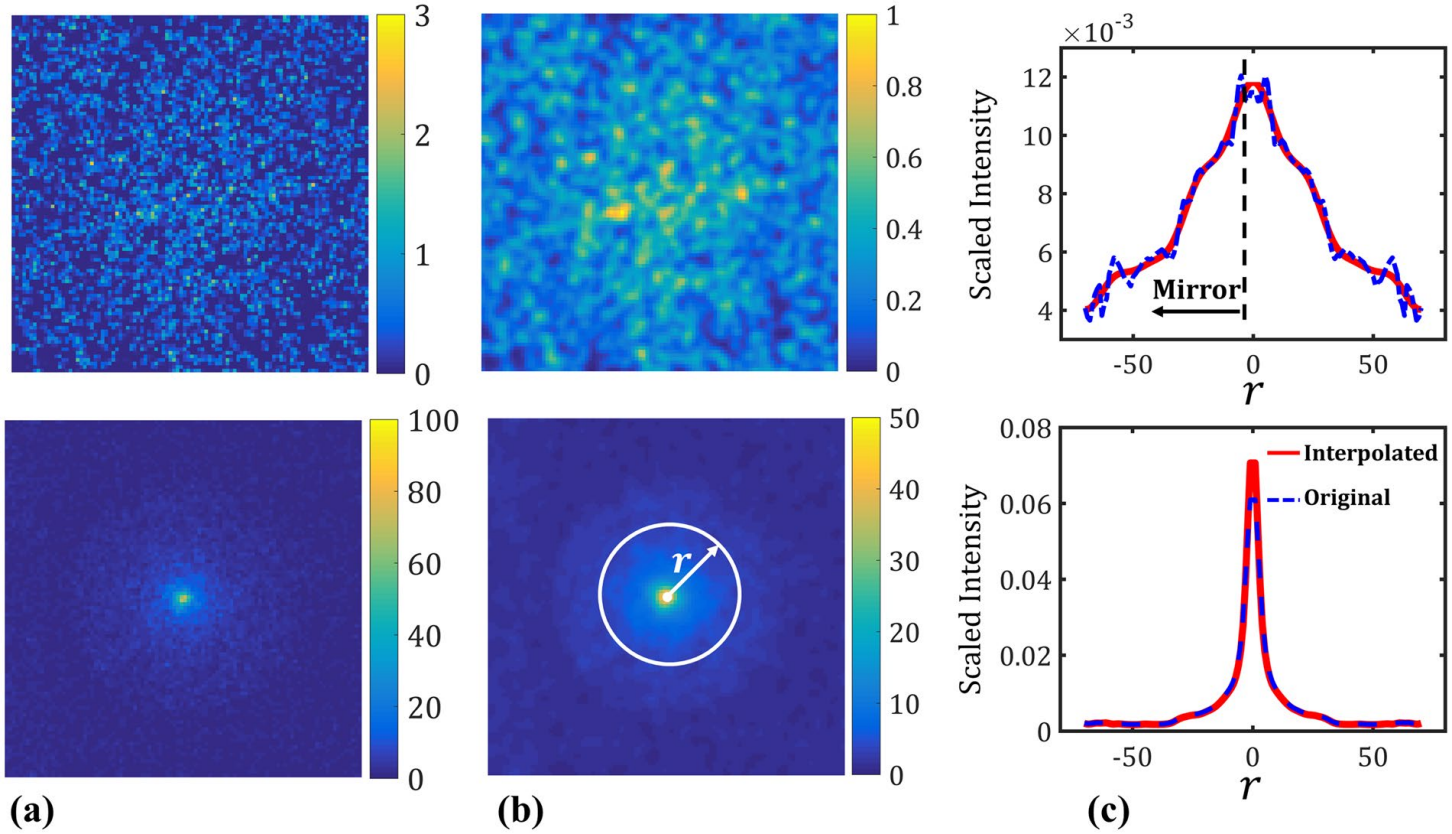
Measuring _*σ*_ for two different images: (**a**) The original scattering patterns obtained by *Zemax* following the schematic in Fig. 1. Both images have a side length of 100 pixels. The simulation settings (i.e., ${\boldsymbol{x}}=[t,\,g,\,{s}_{l}]$) for the images on the top and bottom row correspond to $[51.1,\,0.75,\,0.04]$ and $[24.4,\,0.83,\,0.1]$, respectively. The color bars indicate the range of gray intensity for each image. (**b**) The filtered images. The same Gaussian kernel is used to filter out the local noises in both images in panel (**a**). (**c**) The scaled radial distribution of gray intensity. The area under the dashed blue curve is approximately unity in both cases and the solid red curves are the regressors fitted to the original data. While in the top plot the noise is considerable (even after filtering with a Gaussian kernel), in the bottom plot the noise is negligible and hence the regressor almost interpolates. Gaussian processes are employed as regressors here with an estimated nugget of 10^−3^ and 10^−5^ for the top and bottom plots, respectively.

Measuring *p* for an image is straightforward as it only requires integrating the gray intensity over all the image pixels. Measuring *σ*, however, requires some pre-processing because the amount of scattering in an image is sensitive to noise and has a strong positive correlation with it (i.e., high scattering would involve a high degree of noise in the image and vice versa). As illustrated in Fig. 2, we take the following steps to measure *σ* for an image:

1. Filtering the image with a Gaussian kernel to eliminate the local noises (see panel b in Fig. 2). In general, the width of the Gaussian kernel depends on the resolution of the original image as well as the amount of noise. In our case, the filtering was conducted (in the frequency space) with a kernel width of 7 pixels.
2. Obtaining the radial distribution of the intensity by angularly averaging it over the image.
3. Mirroring the radial distribution to obtain a symmetric curve and then scaling it so that the area under the curve equals unity (see panel c in Fig. 2). At this point, the resulting symmetric curve would approximate a zero-mean Gaussian probability distribution function (PDF).
4. Fit a regressor to further reduce the noise and enrich the scattered data which resemble a Gaussian PDF (compare the solid and dashed lines in panel c).
5. Estimate the standard deviation of the Gaussian PDF via the enriched data. Divide this standard deviation by the power of the image (i.e., *p*) to obtain *σ*.

As for the regressor, we recommend employing a method that can address the *potential* high amount of noise in some of the images which, as mentioned earlier, happens when scattering is significant (e.g., when *t* is large while *g* and ${s}_{l}$ are small). We have used Gaussian processes (GP’s), neural networks, and polynomials for this purpose but recommend the use of GP’s mainly because they, following the procedure outlined in ref.^29^, can automatically address high or small amounts of noise. Additional attractive features of GP’s are discussed in Sec. 2.1 and Sec. 5.

The reason behind scaling the standard deviation in step 5 by *p* is to leverage the negative correlation between the transmitted power and the noise to arrive at a better measure for estimating *scattering*. To demonstrate this, consider two images where one of them is noisier than the other. It is obvious that the noisier image must be more scattered and hence have a larger scattering measure. To increase the difference between the scattering measures (and, subsequently, increase the predictive power of the supervised learner), one can divide them with a variable that is larger (smaller) for the smaller (larger) scattering measure. This variable, in our case, is the transmitted power which is rather robust to the noise.

Finally, we note that the images were not directly used in the supervised learning stage as outputs because: (*i*) Predicting the scattering pattern is not our only goal. Rather, we would like to have a limited set of parameters (i.e., outputs) that can sensibly characterize the image and hence provide guidance as to how the inputs (i.e., $[g,\,{s}_{l},\,t]$) affect the outputs (and correspondingly the scattering patterns). Using the images directly as outputs is a more straightforward approach but renders monitoring the trends difficult. (*ii*) With $100\times 100$ outputs (the total number of pixels), fitting a multi-response supervised learner becomes computationally very expensive and, more importantly, may face severe numerical issues. One can also fit $100\times 100$ single-response supervised learners but this is rather cumbersome, expensive, and prone to errors due to high amounts of noise in some pixels. (*iii*) With $100\times 100$ outputs, the inverse optimization processes (for estimating *g* and ${s}_{l}$ given *t* and an image) becomes expensive.

### Supervised learner: Linking Scattering Patterns with Tissue Sample Characteristics

With the advances in computational capabilities, supervised and unsupervised learning methods have drawn considerable attention in a wide range of applications including computational materials science^30–51^, neuroscience^52^, clinical medicine^53–56^, biology^57,58^, protein analysis and genetics^59^, biotechnology^60,61^, robotics^62^, psychology^63^, climatology^64^, paleoseismology^65^, and economics^66^. These methods provide the means to predict the response of a system where no or limited data is available. Neural networks, support vector machines, decision trees, Gaussian processes (GP’s), clustering, and random forests are amongst the most widely used methods. In case of biological tissues, supervised learning via neural networks has been previously employed, e.g., for classification of tissues using SFDI-based training datasets^67–72^.

We employ GP’s to link the characterizing parameters of the scattering patterns (i.e., *p* and *σ*) with those of the tissue samples (i.e., *t*, *g*, and ${s}_{l}$). Briefly, the essential idea behind using GP’s as supervised learners is to model the input-output relation as a realization of a Gaussian process. GP’s are well established in the statistics^73^, computational materials science^33,40^, and computer science^74^ communities as they, e.g., readily quantify the prediction uncertainty^75,76^ and enable tractable and efficient Bayesian analyses^77,78^. In addition, GP’s are particularly suited to emulate highly nonlinear functions especially when insufficient training samples are available.

In our case, the inputs and outputs corresponds to ${\boldsymbol{x}}=[t,\,g,\,{s}_{l}]$ and ${\boldsymbol{y}}=[p,\,\sigma ]$, respectively. As there are two outputs, we can either fit a multi-response GP (MRGP) model or two independent single-response GP (SRGP) models. With the former approach, one GP model is fitted to map the three-dimensional (3*D*) space of ***x*** to the two-dimensional (2*D*) space of ***y***. With the latter approach, however, two GP models are fitted: one for mapping ***x*** to *p* and another for mapping ***x*** to *σ*. The primary advantage of an MRGP model lies in capturing the correlation between the responses (if there is any) and, subsequently, requiring less data for a desired level of accuracy. An MRGP model might not provide more predictive power if the responses are independent, have vastly different behavior, or contain different levels of noise.

**Figure 3:**
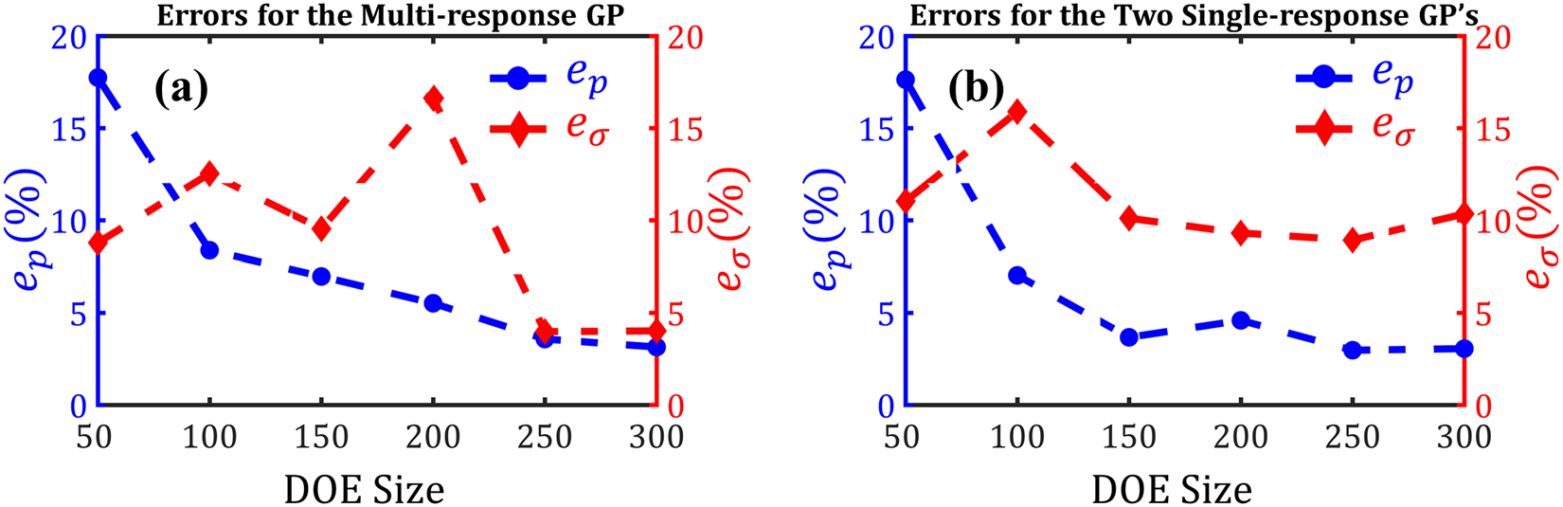
Reduction of prediction error (in percent) by increasing the number of training samples: (**a**) Prediction errors for the MRGP model. (**b**) Prediction errors for the SRGP models. The errors are all calculated based on 100 test points that were not used in fitting.

We conducted convergence studies to decide between the two modeling options and, additionally, determine the minimum DOE size required to fit a sufficiently accurate model. As mentioned earlier, the Sobol sequence was employed to build one DOE of size 400 over the hypercube $0.7\le g\le 0.93\,$, $0.03\le {s}_{l}\le 0.12\,mm$, and $200\le t\le 600\,\mu m$. Sobol sequence was chosen over other design methods (e.g., Latin hypercube) because consecutive subsets of a Sobol sequence all constitute space-filling 50 designs. Following this, we partitioned the first 300 points in the original DOE of size 400 into six subsets with an increment of 50, i.e., the ${i}^{th}$ DOE (*i* = 1,…, 6) included points $1,\,\ldots ,i\times 50\,$from the original DOE. The last 100 points in the original DOE (which are space-filling and different from all the training points) were reserved for estimating the predictive power of the models. Next, three GP models were fitted to each DOE: (*i*) an MRGP model to map ***x*** to ***y***, and (*ii*) two SRGP models; one to map ***x*** to *p* and another to map ***x*** to *σ*. Finally, the reserved 100 DOE points were used to estimate the scaled root-mean-squared error (RMSE) as:

2 $${e}_{q}=\sqrt{\frac{1}{N}{\sum _{i=1}^{N}(\frac{q-\hat{q}}{q})}^{2}},$$

where *N* is the number of prediction points (*N* = 100 in our case), *q* is the quantity of interest (either *p* or *σ*), and $\hat{q}$ is the estimated quantity by the fitted model. Figure 3 summarizes the results of our convergence studies (see Sec. 5.1 for fitting costs) and indicates that:

1. As the sample size increases, the errors generally decrease. The sudden increases in the errors are either due to overfitting or the addition of some noisy data points.
2. ${e}_{\sigma }$ of the MRGP model is almost always smaller than that of the SRGP (compare the red curves in Fig. 3a and b). The opposite statement holds for ${e}_{p}$. This is because *p*, as compared to *σ*, is much less noisy.

**Figure 4:**
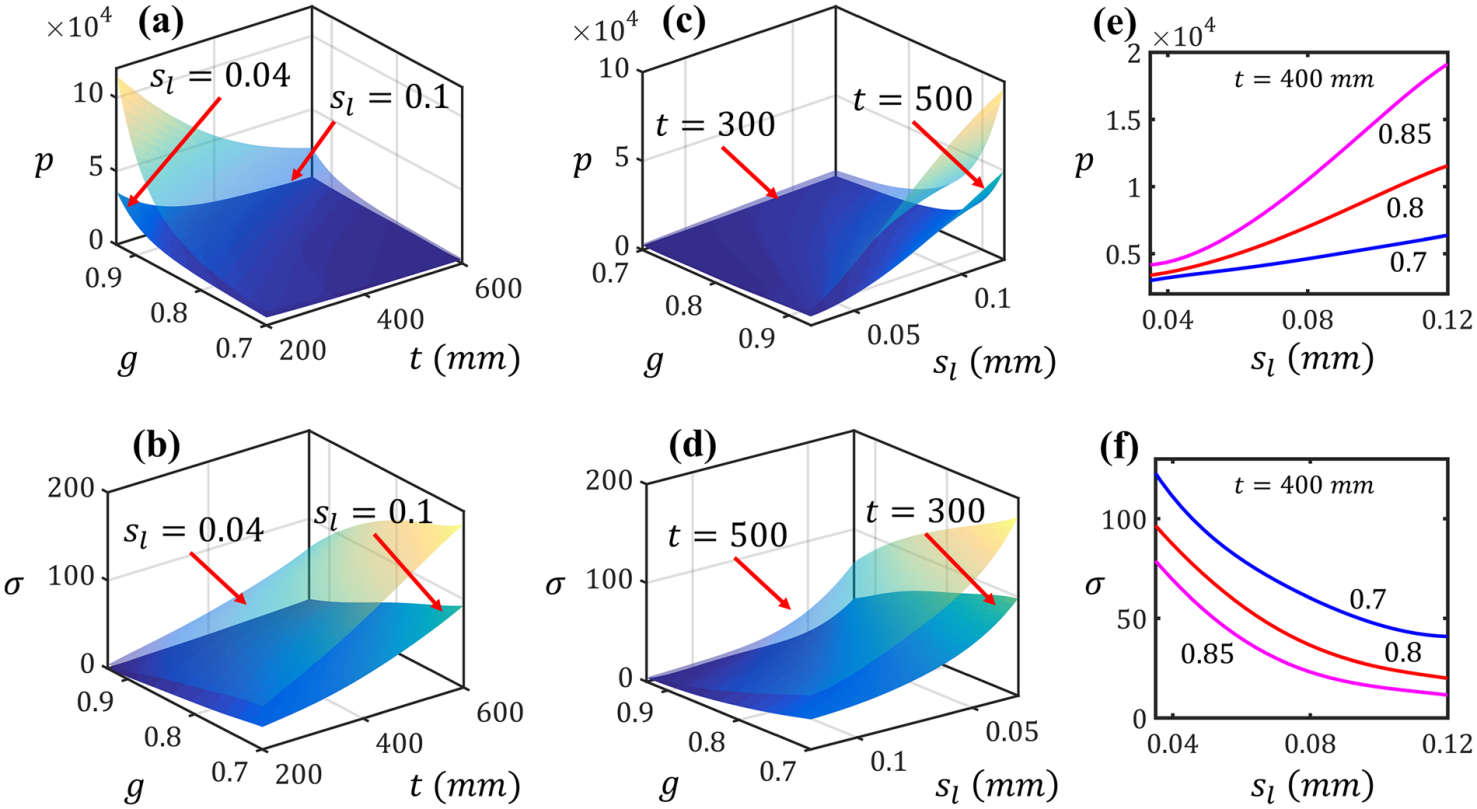
Effect of the characteristics of tissue samples (i.e., $[t,\,g,\,\mu ]$) on the characteristics of scattering patterns (i.e, $[p,\,\sigma ]$): The effect on the transmittted power and the scaled Gaussian width are demostrated in the figures in, respectively, top and bottom rows. In (**a**) through (**d**), either *t* or *μ* is fixed and the outputs are plotted versus the other two inputs. In (**e**) and (**f**), *p* and *σ* are plotted versus *μ* for three values of *g* while having *t* fixed to 400 *mm*.

Based on the convergence studies, we can conclude that an MRGP model with at least 300 training data points can provide, on average, prediction errors smaller than 5%. Following this, we fitted an MRGP model in 28.6 seconds to the entire dataset (i.e., DOE of size 400) and employed it in the subsequent analyses in Sec. 2.2. Figure 4 illustrates how *p* and *σ* (and hence the scattering patterns) change as a function of tissue sample characteristics based on this MRGP model. The plots in top and bottom rows of Fig. 4 demonstrate the effect of inputs on, respectively, the transmittted power and the scaled Gaussian width. In Fig. 4(a) and (b), ${s}_{l}$ is fixed to either $0.1\,mm$ or $0.04\,mm$ and the outputs are plotted versus *t* and *g*. In Fig. 4(c) and (d), *t* is fixed to either 300 *mm*, or 500 *mm* and the outputs are plotted versus *g* and ${s}_{l}$. In Fig. 4(e) and (f), *p* and *σ* are plotted versus ${s}_{l}$ for three values of *g* while having *t* fixed to 400 *mm*. In summary, these plots demonstrate that decreasing a sample’s *g* or ${s}_{l}$, or increasing its thickness, would decrease the transmitted power while increasing the scattering (i.e., *σ*). Moreover, both *p* and *σ* change monotonically as a function of the inputs. This latter feature enables us to uniquely estimate *g* and ${s}_{l}$ given *t*, *p*, and *σ*.

To quantify the relative importance of each input parameter on the two model outputs, we conducted global sensitivity analysis (SA) by calculating the Sobol indices (SI’s)^79,80^. As opposed to local SA methods which are based on the gradient, SI’s are variance-based quantities and provide a global measure for variable importance by decomposing the output variance as a sum of the contributions of each input parameter or combinations thereof. Generally, two indices are calculated for each input parameter of the model: *main* SI and *total* SI^81^. While a main SI measures the first order (i.e., additive) effect of an input on the output, the total SI measures both the first and higher order effects (i.e., including the interactions). SI’ are normalized quantities and known to be efficient indicators of variable importance because they do not presume any specific form (e.g., linear, monotonic, etc.) for the input-output relation.

**Figure 5:**
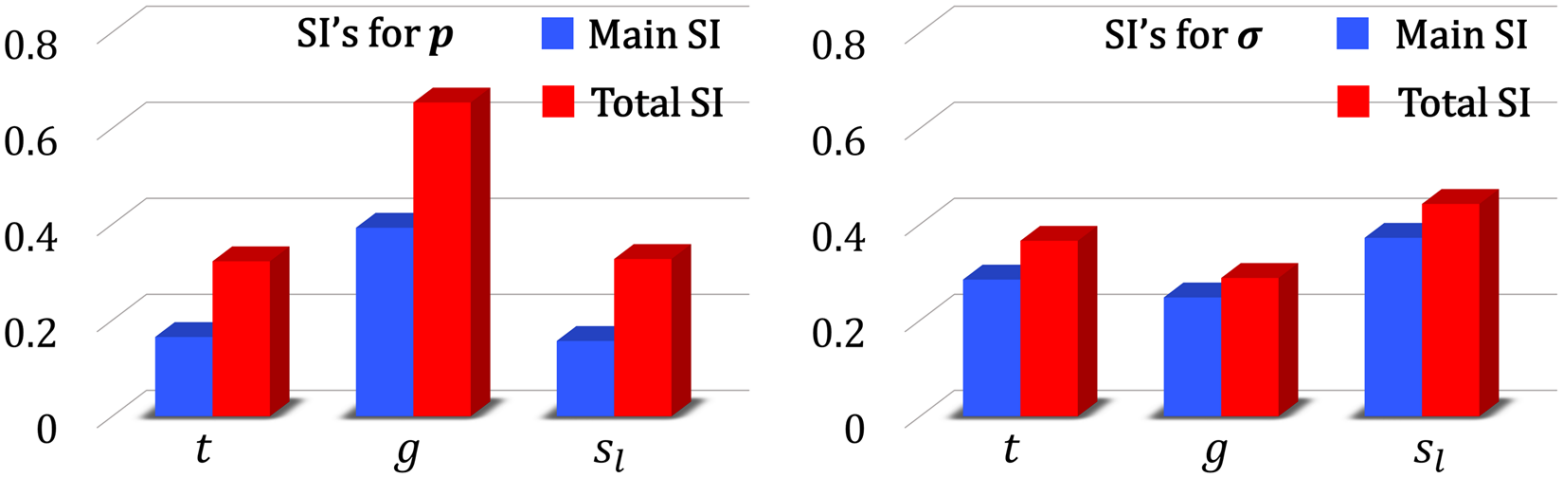
Results of the sensitivity analyses: Main and total sensitivity indices (SI’s) for the transmitted power (left) and the scaled Gaussian width (right).

Using the MRGP model, we conducted quasi Monte Carlo simulations to calculate the main and total SI’s of the three inputs for each of the outputs. The results are summarized in Fig. 5 and indicate that all the inputs affect both outputs. While *p* is noticeably sensitive to *g* (and equally sensitive to *t* and ${s}_{l}$), *σ* is almost equally sensitive to all the inputs. It is also evident (as captured by the difference between the height of the two bars for each input) that there is more interaction between the inputs in the case of *p* than *σ*.

### Inverse Optimization: Estimating the Scattering Properties of a Tissue

Noting that the sample thickness can be controlled in an experiment, tissue characterization is achieved by finding the scattering parameters of the sample given how it scatters a pencil beam in a setup similar to that in Fig. 1. More formally, in our case, tissue characterization requires estimating *g* and ${s}_{l}$ given *p*, *σ*, and the sample thickness *t*. Although in principle we can inverse the MRGP model at any fixed *t* to map $[p,\,\sigma ]$ to $[g,\,{s}_{l}]$, this is rather cumbersome. Hence, we cast the problem as an optimization one by minimizing the cost function, *F*, defined as:

3 $$[\hat{g},\,{\hat{s}}_{l}]=\mathop{{argmin}}\limits_{g,\,{s}_{l}}\,F=\mathop{{argmin}\,{log}}\limits_{g,\,{s}_{l}}\,({(1-\frac{{p}_{GP}}{{p}_{e}})}^{2}+{(1-\frac{{\sigma }_{GP}}{{\sigma }_{e}})}^{2}+1),$$

where *F* is the cost function which measures the difference between the experimental values (i.e., ${p}_{e}$ and ${\sigma }_{e}$) and the predicted ones by the MRGP model (i.e., ${p}_{GP}(t,g,\,{s}_{l})$ and ${\sigma }_{GP}(t,g,\,{s}_{l})$). We note that, the model predictions are subject to $t={t}_{e}$ where ${t}_{e}$ is the sample thickness.

**Figure 6:**
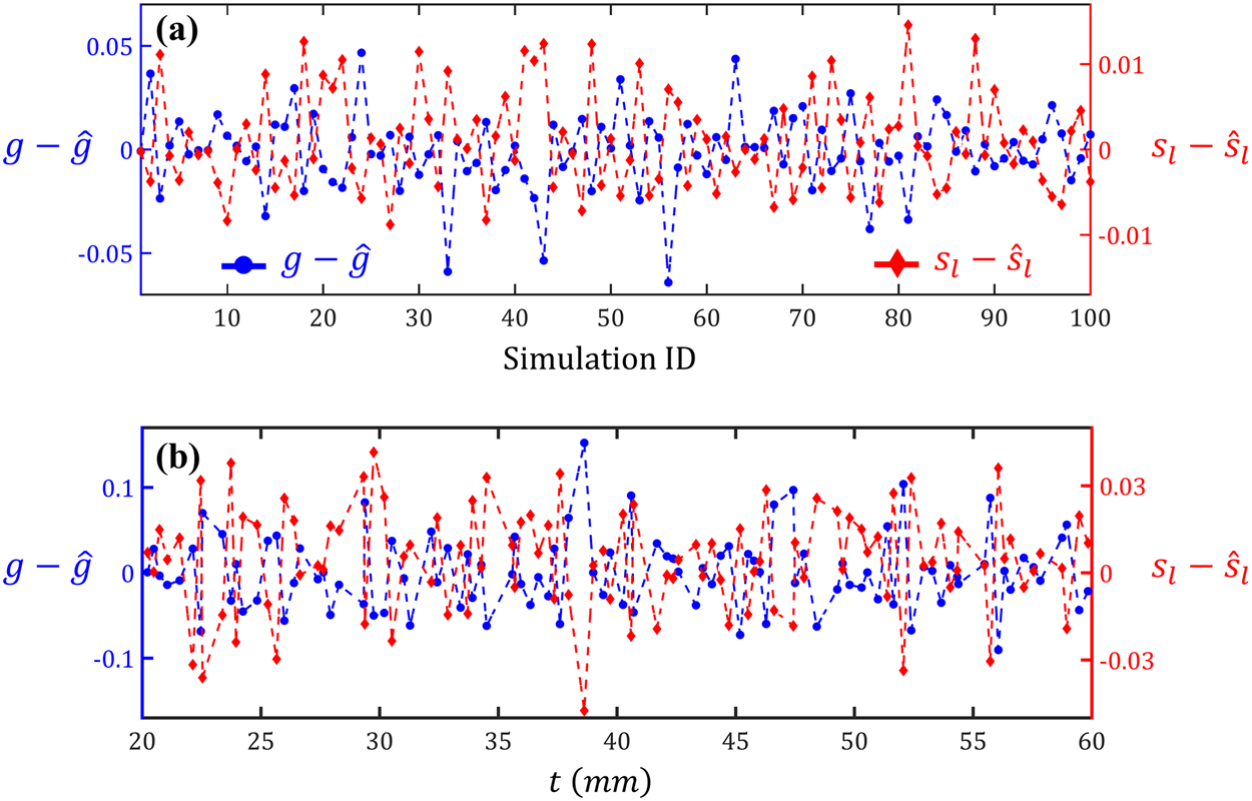
Errors in predicting the scattering properties of 100 tissue samples: The scattering properties of most samples have been estimated quite accurately. In (**a**) and (**b**) the simulations are ordered with respect to, respectively, simulation number and the sample thickness.

To test the accuracy of the fitted MRGP model in estimating the scattering parameters, we generated a space-filling test dataset of size 100 while ensuring that none of the test points were the same as the 400 training ones used in fitting the MRGP. For each test point, then, the outputs (i.e., *p* and *σ*) and the sample thickness (i.e., *t*) were used to estimate the inputs (i.e., $\hat{g}$ and ${\hat{s}}_{l}$) by minimizing Eq. 3. To solve Eq. 3, we used the $Fmincon$ command in the optimization toolbox of MATLAB®. Figure 6(a) illustrates the prediction errors of estimating *g* (on the left axis) and ${s}_{l}$ (on the right axis) for the 100 test points. It is evident that the average errors are zero in estimating either *g* or ${s}_{l}$, indicating that the results are indeed unbiased. In Fig. 6(b) the errors are plotted with respect to the sample thickness to investigate whether they are correlated with *t*. As no obvious pattern can be observed, it can be concluded that our procedure for estimating *g* and ${s}_{l}$ is quite robust over the range where *t* is sampled in the training stage.

**Figure 7:**
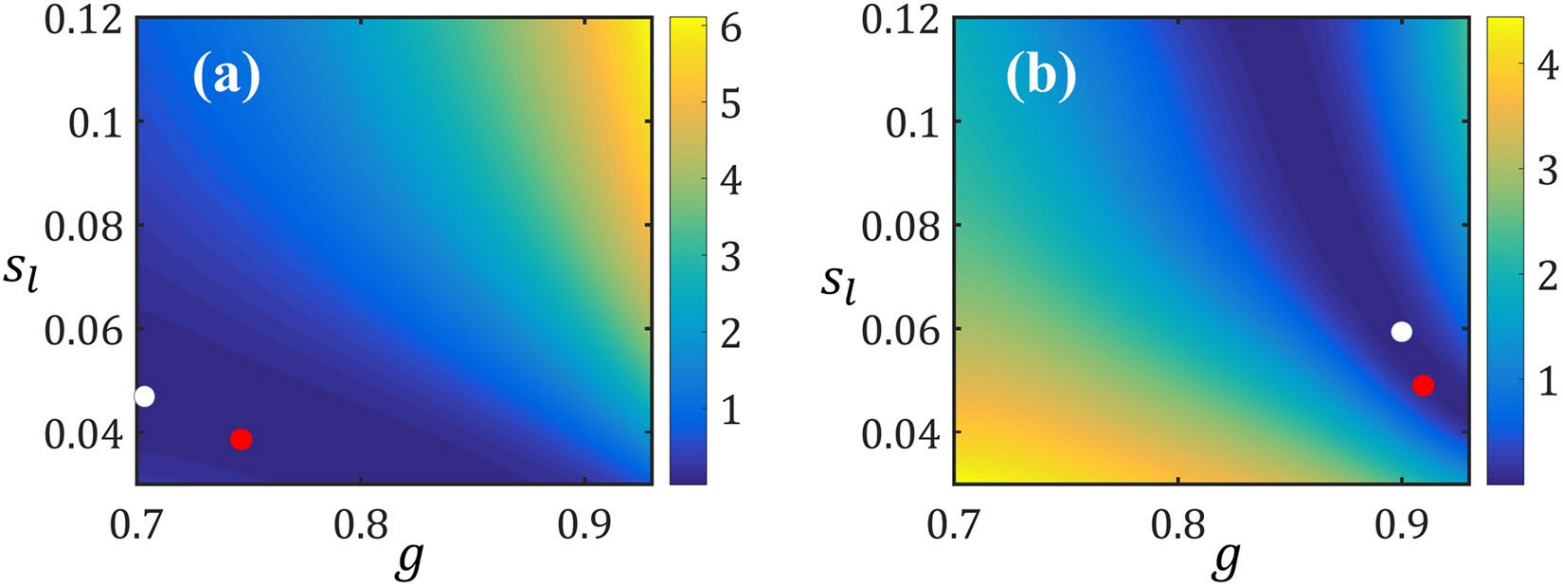
Contours of *F* as a function of the optimization variables: The fixed variables are $[t,\,p,\,\sigma ]=[31.3,\,4.2\times {10}^{3},\,70.1]$ in (**a**) and $[t,\,p,\,\sigma ]=[25.7,\,2.7\times {10}^{4},\,8.3]$ in (**b**). The true and estimated optima are indicated with, respectively, white and red dots in each case.

For further investigations, we normalize the errors and provide the summary statistics in Table 1. As quantified by the scaled RMSE, on average, the prediction errors are relatively small, especially for the *g*. The maximum *scaled* errors are $9.1 \%$ (corresponding to simulation ID 56 in Fig. 6) and $18.1 \%$ (simulation ID 22) for *g* and ${s}_{l}$, respectively. Figure 7 demonstrates the contour plots of the cost function for these two simulations. As it can be observed, in each case there are regions in the search space of $[g,\,{s}_{l}]$ where the cost function *F* is approximately constant. In fact, the true optimum and the estimated solution (indicated, respectively, with white and red dots in Fig. 7) are on the loci where *F* is minimized. The existence of such loci can be explained by noting that *g* and ${s}_{l}$ have similar effects on both responses, i.e., increasing (decreasing) either of them would decrease (increase) *σ* while increasing (decreasing) *p*.

**Table 1:** Summary of prediction errors: The scaled RMSE (see Eq. 2) and scaled maximum error are calculated for the 100 data points in Fig. 6.

	Scaled RMSE (%)	Scaled Max. Error (%)
***g***	2.41	9.1
***s***_***L***_	7.87	18.1

Finally, we note that the inverse optimization cost is negligible (less than 10 seconds) in our case because we have employed a gradient-based optimization technique which converges fast because (*i*) it uses the predictions from the MRGP model for both the response and its gradient (which are done almost instantaneously), and (*ii*) we have reduced the dimensionality of the problem from 100 × 100 (the number of pixels in each image) to two (i.e., $[p,\,\sigma ]$).

## Discussions

In our computational approach, the accuracy in predicting the scattering parameters of a turbid medium mainly depends on (*i*) the errors in *Zemax* simulations, (*ii*) the predictive power of *p* and *σ* in characterizing the scattering patterns, and $(iii)$ the effectiveness of the supervised learner and the optimization procedure.

The inherent numerical errors in *Zemax* inevitably introduce some error into the training dataset. In addition, the number of launched rays in our Monte Carlo simulations, though having utilized the maximum capacity of *Zemax*, might be insufficient and hence introduce some inaccuracies. This latter source of error particularly affects samples which scatter the incoming LED beam more (e.g., thick samples with small *g* and ${s}_{l}$) because once the number of segments per a launched ray exceeds the software’s limit, the ray is discarded.

To reduce the problem dimensionality and enable the supervised learning process, the images of the scattering patterns (see, e.g., Fig. 2a) were characterized with two negatively correlated parameters, namely, *p* and *σ*. Since the images are not entirely symmetric and may not completely resemble a Gaussian pattern, employing only *σ* to capture their patterns’ spread will introduce some error. We have addressed this source of error, to some extent, by filtering the images with a Gaussian kernel (see, e.g., Fig. 2b) and enriching the radial distribution of the scattering patterns by a GP regressor (see Fig. 2c). Our choice of regressor, in particular, enabled automatic filtering of small to large amounts of noise through the so-called *nugget parameter*. Additionally, we leveraged the negative correlation between *p* and *σ* in the definition of *σ* to increase its sensitivity to the spreads. The supervised learning and inverse optimization procedures will, of course, benefit from reducing the simulation errors and finding parameters with more predictive power than *σ*.

As for the supervised learner, we illustrated that a multi-response Gaussian process can provide sufficient accuracy with a relatively small training dataset (see Fig. 3a). Learning both responses (i.e., *p* and *σ*) simultaneously, in fact, helped to better address the noise due to the negative correlation between the responses. As demonstrated in Fig. 3a, the MRGP model with 300 training samples can achieve, on average, errors smaller than 5%. Increasing the size of the training dataset would decrease the error but, due to the simulation errors, an RMSE of zero cannot be achieved. Additionally, sensitivity analyses were conducted by calculating the Sobol indices of the inputs (i.e., *t*, *g*, and)using the MRGP model. As illustrated in Fig. 5, all the inputs are effective and affect both outputs with *p* being noticeably sensitive to *g* and embodying more interactions between the inputs.

We casted the problem of determining the scattering parameters as an inverse optimization one where *g* and ${s}_{l}$ of a tissue sample were estimated given its thickness *t*, and the corresponding scattering pattern (i.e., *p* and *σ*). In optimization parlance, the objective or cost function (defined in Eq. 3) achieves the target scattering pattern by searching for the two unknown inputs while constraining the sample thickness. As illustrated in Fig. 6 for 100 test cases, our optimization procedure provides an unbiased estimate for the scattering parameters with an error of roughly 5%. The inaccurate estimations in our optimization studies are because *g* and ${s}_{l}$ have similar effects on *p* and *σ*. This is demonstrated in Fig. 7 where the local optima of the objective function create a locus and hence overestimating *g* or ${s}_{l}$ would result in underestimating the other and vice versa. To quantify this effect, we calculated Spearman’s rank-order correlation between the errors $g-\hat{g}$ and $\mu -{\hat{s}}_{l}$ for the 100 data points reported in Fig. 6 and found it to be $-0.90$. Such a strong negative correlation value (Spearman’s rank-order correlation is, in the absence of repeated data values, between $-1$ and 1) indicates that when *g* is underestimated, ${s}_{l}$ will be overestimated and vice versa.

**Figure 8:**
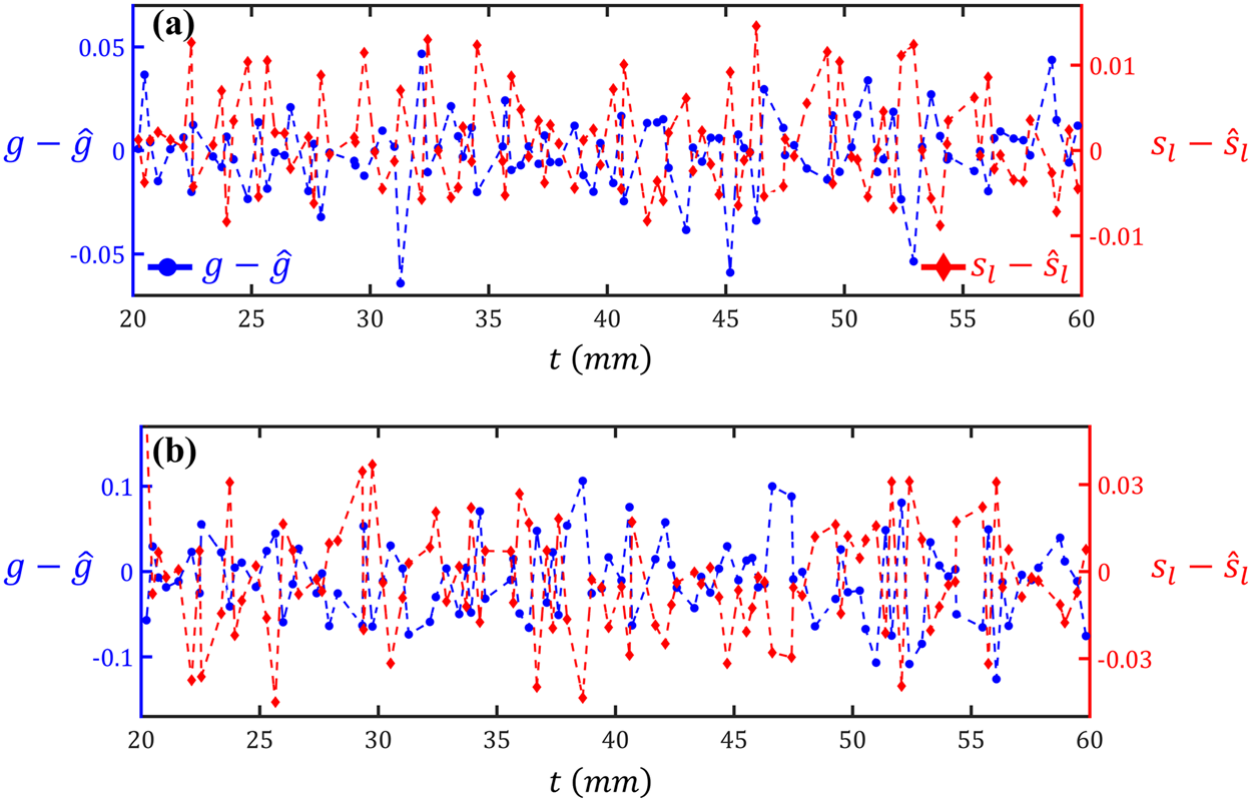
Errors in predicting the scattering properties of 100 tissue samples while enforcing 10% error in thickness value: In (**a**) and (**b**) the thickness value used in the inverse optimization is, respectively, increased and decreased by 10% from the original value used in *Zemax* simulations. The simulations are ordered with respect to the sample thickness.

When coupling our method with experimental data, there will be some measurement errors primarily due to the noise of the camera sensor, insufficient rejection of the un-scattered waves, and the inaccuracy in determining the sample thickness. To address the dark current noise of the camera, the camera integration time should be increased. It is also favorable to increase the power of LED to reduce the influence of the parasitic rays of the environment, but caution must be practiced to avoid saturating the camera or damaging the sample. To ensure proper rejection of the un-scattered rays, an image corresponding to the absolute pixel-by-pixel difference of the two images with and without the polarization filter should be obtained. Then, the maximum intensity on the difference image should be compared with that of the image obtained with the polarization filter. Lastly, to minimize the errors due to sample thickness, samples with uniform and carefully measured thickness must be prepared. Inaccuracies in measuring the sample thickness or using considerably non-uniform ones, will adversely affect the prediction results. To quantify the sensitivity of the predictions to inaccuracies associated with thickness, we repeated the inverse optimization process in Sec. 2.2 considering potential measurement errors of 10%. In particular, we redid the inverse optimization for the test dataset while employing thickness values with 10% difference from the true values (i.e., instead of *t*, $0.9t$ or $1.1t$ were used). The estimated values (i.e., $\hat{g}$ and ${\hat{s}}_{l}$) where then compared to the true ones. As summarized in Table 2, the scaled RMSE’s have, especially in the case of ${s}_{l}$, increased. To see whether the sample thickness has a correlation with the errors, in Fig. 8 the errors are plotted versus *t*. Although the errors are quite large in some cases (which is expected because the true *t* value is not used in the inverse optimization), the overall results are unbiased (i.e., the average errors are close to zero). These results indicate that, as long as the sample thickness is measured sufficiently accurate, the model can provide an unbiased estimate for *g* and ${s}_{l}$.

**Table 2:** Summary of prediction errors while enforcing 10% error in thickness: The scaled RMSE’s (see Eq. 2) are calculated for the 100 test points while enforcing 10% difference between the sample thickness used in the simulations and the thickness used in the inverse optimization.

	Errors with 10% Overestimation of *t*	Errors with 10% Underestimation of ***t***
***g***	$5.90$	$5.32$
*s*_***L***_	$33.02$	$28.11$

Lastly, we compare the accuracy of our approach to other methods. The reported errors in Fig. 3, Table 1, and Fig. 6 do not consider the errors that will be introduced upon experimental data collection. As explained below, our error estimates are comparable to those of the FDPM, SFDI, and IAD methods from a computational standpoint. It is noted that in all these methods (including ours) the dominating error will be associated with experimental data (once it is used in conjunction with simulations).

In FDPM, the model bias^77^ originates from the assumptions made for solving the diffusion equation (e.g., using a semi-infinite medium as opposed to a finite-size sample)^82^. Besides this, there are two other error sources in FDPM (*i*) the preliminary error due to approximating light transport in tissues with diffusion equation is estimated to be 5~10%^83^, and (*ii*) the error due to the quantum shot noise limit of the instrument which depends on the configuration and components of the system. For a reasonable system comprising of two detectors and one source at the modulation frequency of $500\,MHz$, the limit of quantum shot noise results in about 2% error in estimating the scattering coefficient^82^. The scattering length, ${s}_{l}$, is the inverse of the scattering coefficient and will roughly have the same error. Similarly, the percentage errors of ${s}_{l}$ roughly equals to that of the reduced scattering coefficient defined as $(1-g)/{s}_{l}$. Considering only these two sources of noise, we can assume a total noise of around 10% for the FDPM technique. As for the SFDI technique, the diffusion approximation results in an overall reported error of around 3% for the reduced scattering coefficient^9^. In the IAD method, the prediction errors are sensitive to the input data. For instance, it is reported that with a 1% perturbation in the inputted transmission and reflection amounts, the relative error in estimating the scattering coefficient and anisotropy factor increases 10 and 4 times, respectively^13^.

## Conclusion

We have introduced a non-invasive method for computational characterization of the scattering parameters (i.e., the anisotropy factor and the scattering length) of a medium. The essence of our approach lies in finding a supervised learner that can predict the scattering pattern of a turbid medium given its thickness and scattering parameters. Once this supervised learner is found, we solve an inverse optimization problem to estimate the scattering parameters of any turbid sample given its thickness and the image of the scattered rays’ pattern. Additionally, our approach is computationally inexpensive because the majority of the cost lies in building the training dataset which is done once.

To the best of our knowledge, this is one of the simplest and most inexpensive methods of tissue characterization because, in practice, only a few basic and low-cost instruments such as an LED, an aperture, a polarizer, and a camera are required. Additionally, our analyses and results are independent of the wavelength of the LED and therefore the scattering parameters of many tissues can be estimated over a wide range of visible and infrared wavelengths. We note that, in our method it is assumed that the absorption is much weaker than the scattering and thus its effect on the output images is negligible. This assumption holds for some tissues including white brain matter, grey brain matter, cerebellum, and brainstem tissues where the scattering coefficient is more than 100 times larger than the absorption coefficient in most of the visible and in the reported near-infrared range^27^ (see Table 3 in ref.^84^ for more details). Measuring weak absorption of tissue with our method requires more intense data analysis and processing. However, we believe that this limit doesn’t translate into impracticality of our method as there are methods^22,23^ which can only estimate *g* and ${s}_{l}$.

We plan to experimentally validate our approach and quantify the effect of measurement errors (due to, e.g., the noise of the camera sensor and insufficient rejection of the un-scattered waves) on estimating the scattering parameters. We believe that this method has the potential to facilitate the fabrication of tissue phantoms used for diagnostic and therapeutic purposes over a wide range of optical spectrum.

## Methods

### Gaussian Process Modeling

GP modeling has become the de-facto supervised learning technique for fitting a response surface to training datasets of either costly physical experiments or expensive computer simulations due to its simplicity, flexibility, and accuracy^74,85–88^. The fundamental idea of GP modeling, is to model the dependent variable, *y*, as a realization of a random process, *Y*, with inputs ${\boldsymbol{x}}=[{x}_{1},\,{x}_{2},\,\ldots ,\,{x}_{n}]$ where $n\in {\mathbb{N}}$ is the number of inputs. The underlying regression model can be formally stated as:

4 $$Y({\boldsymbol{x}})=\sum _{i=1}^{k}{\beta }_{i}\,{f}_{i}({\boldsymbol{x}})+Z({\boldsymbol{x}})$$

where ${\boldsymbol{f}}({\boldsymbol{x}})=[{f}_{1}({\boldsymbol{x}}),\,\ldots ,\,{f}_{k}({\boldsymbol{x}})]$ are a set of known basis functions, ${\boldsymbol{\beta }}=[{\beta }_{1},\,\mathrm{..},\,{\beta }_{k}]$ are unknown coefficients, and $Z({\boldsymbol{x}})$ is the random process. In Eq. 4, following the statistical conventions, the random variables/processes and their realizations are denoted with, respectively, upper and lower cases. Assuming $Z({\boldsymbol{x}})$ is a zero-mean GP with covariance function $c(\cdot ,\cdot )$ of the form

5 $$c({\boldsymbol{x}},{\boldsymbol{w}})=sR({\boldsymbol{x}},{\boldsymbol{w}})$$

between $Z({\boldsymbol{x}})$ and $Z({\boldsymbol{w}})$, GP modeling essentially consists of estimating the ***β*** coefficients, process variance *s*, and parameters of the correlation function $R(\cdot ,\,\cdot )$. Often, the maximum likelihood estimation (MLE) method is used for this purpose^89,90^.

We implemented an in-house GP modeling code in Matlab® following the procedure outlined in ref.^29^. The so-called Gaussian correlation function was employed with an addition of a nugget parameter, *δ*, to address the possible noises:

6 $$R({\boldsymbol{x}},{\boldsymbol{w}})=\exp (-\sum _{i=1}^{n}{\theta }_{i}{({x}_{i}-{w}_{i})}^{2})+\delta ,$$

where ***θ*** are the roughness parameters estimated via MLE. For noiseless datasets, *δ* is generally set to either a very small number (e.g., 10^−8^) to avoid numerical issues, or zero. In our work, we have used GP’s for two purposes: (*i*) to smooth out the radial distribution of the scattered rays and enrich the associated PDF for a better estimation of its standard deviation (see Fig. 2), and (*ii*) to fit a response surface for mapping ${\boldsymbol{x}}=[t,\,g,\,\mu ]$ to ${\boldsymbol{y}}=[p,\,\sigma ]$. We emphasize that, the adaptive procedure of ref.^29^ allows to adjust *δ* in Eq. 6 to address negligible to large amounts of noise.

Multiple studies have extended GP modeling to multi-output datasets. Of particular interest, has been the work of Conti. *et al*.^91^ where the essential idea is to concatenate the vector of responses (i.e., ${\boldsymbol{y}}={[{{\boldsymbol{y}}}_{1},\ldots ,{{\boldsymbol{y}}}_{u}]}^{T}$ for *u* outputs) and model the covariance function as $c({\boldsymbol{x}},{\boldsymbol{w}})={\boldsymbol{s}}\otimes R({\boldsymbol{x}},{\boldsymbol{w}})$ where ${\boldsymbol{s}}$ is the $u\times u$ covariance matrix of the responses and ⊗ is the Kronecker product. Finally, it is noted that since we did not know *a priori* how $p$ and $\sigma$ change as a function of ${\boldsymbol{x}}=[t,\,g,\,\mu ]$, a constant basis function was used (i.e., $f({\boldsymbol{x}})=1$) in all our simulations.

The computational cost of fitting each of the MRGP models used in the convergence study is summarized in Table 3. As it can be observed, the costs are all small and increase as the size of the training dataset increases.

**Table 3:** Computational cost of fitting MRGP models in our convergence study: As the number of the training samples increases, the fitting cost increases as well.

	Model 1	Model 2	Model 3	Model 4	Model 5	Model 6
Training Size	$50$	$100$	$150$	$200$	$250$	$300$
Fitting Cost (seconds)	$1.83$	$3.68$	$5.89$	$8.00$	$14.00$	$16.89$

### Sensitivity Analysis with Sobol Indices

Sobol indices (SI’s) are variance-based measures for quantifying the global sensitivity of a model output to its inputs. For a model of the form $y({\boldsymbol{x}})=f({x}_{1},\,\ldots ,\,{x}_{n})$, the main SI for the ${i}^{th}$ input ${x}_{i}$ is calculated as:

7 $${S}_{i}=\frac{{V}_{{X}_{i}}({E}_{{{\boldsymbol{X}}}_{ \sim i}}(Y|{X}_{i}))}{V(Y)},$$

where $V(Y)$ is the total variance of the output, ${{\boldsymbol{X}}}_{ \sim i}$ denotes all the inputs except ${X}_{i}$, ${V}_{{X}_{i}}$ is the variance with respect to ${x}_{i}$, and ${E}_{{{\boldsymbol{x}}}_{ \sim i}}(Y|{x}_{i})$ is the expectation of *Y* for all the possible values of ${{\boldsymbol{X}}}_{ \sim i}$ while keeping ${X}_{i}$ fixed. Using the law of total variance, one can show that ${S}_{i}$’s are normalized quantities and vary between zero and one. It is noted that, similar to the above, the random variables and their realizations are denoted with, respectively, upper and lower cases.

The total SI for the ${i}^{th}$ input is calculated as:

8 $${S}_{{T}_{i}}=\frac{{E}_{{{\boldsymbol{X}}}_{ \sim i}}({V}_{{X}_{i}}(Y|{{\boldsymbol{X}}}_{ \sim i}))}{V(Y)}=1-\frac{{V}_{ \sim {{\boldsymbol{X}}}_{i}}({E}_{{X}_{i}}(Y|{{\boldsymbol{X}}}_{ \sim i}))}{V(Y)}$$

which includes the contributions from all the terms in the variance decomposition that include ${X}_{i}$. Comparing Eqs 7 and 8, it is evident that $0\le {S}_{i}\le {S}_{{T}_{i}}\le 1$. Saltelli *et al*.^81^ provided numerical methods based on quasi Monte Carlo simulations for efficiently calculating both the main and total SI’s.

### Data availability

The datasets and statistical models generated during this study are available from the corresponding author.
